# Large Tracheocutaneous Fistula Successfully Treated With Bronchoscopic Intervention and Flap Grafting: A Case Report and Literature Review

**DOI:** 10.3389/fmed.2020.00278

**Published:** 2020-06-23

**Authors:** He-huan Li, Ning Zhao, Jing-wen Lu, Ru-rong Tang, Chao-nan Liang, Gang Hou

**Affiliations:** ^1^Department of Breast Surgery, First Hospital of China Medical University, Shenyang, China; ^2^Department of Otolaryngology, First Hospital of China Medical University, Shenyang, China; ^3^Department of Pulmonary and Critical Care Medicine, First Hospital of China Medical University, Shenyang, China; ^4^Department of Anesthesiology, First Hospital of China Medical University, Shenyang, China

**Keywords:** tracheocutaneous fistula, bronchoscopy, airway stent, flap grafting, degradable materials

## Abstract

Tracheocutaneous fistula (TCF) is the most common related post-operative complication after tracheotomy. Treatments such as surgery and flap grafting are usually applied to close TCFs. We report a case of a large TCF with an area of ~3.0 cm × 1.0 cm. Here, we describe a relatively convenient approach for the management of a patient with a large TCF. In our treatment strategy, a coverd tracheal stent was used to cover the defect by bronchoscopy, the bronchial defect was closed with a local turnover flap, the structure was reinforced with biodegradable material (RapidSorb Plate 2.0), and then transplantation of a deltopectoral flap was performed. It is worth noting that the patency of the trachea was maintained during the whole surgery course. No recurrence or complications occurred after the 12-month follow-up. The large TCF was successfully treated with bronchoscopic intervention, biodegradable material and flap grafting, and without cartilage grafting.

## Introduction

Tracheocutaneous fistula (TCF) is usually related to long-term tracheostomy, chronic infection, chronic granulomatosis and post-operative radiotherapy treatment. Above all, prolonged tracheostomy is the most common cause of TCFs (1). Patients who previously suffered from thyroid carcinoma and underwent surgical resection also more easily develop TCFs (2). Various methods for the closure of a large TCF with surgery and flap grafting have been described, including the use of two overlapping skin flaps (3), turnover hinge flap, V-Y advancement flap (4), prefabricated radial forearm free flap (5), pregrafted palatal mucosa (6), Z-plasty technique (7), and perichondrial flap graft (8).

The closure of a small TCF is often simply, safely, and successfully accomplished by performing limited local procedures, whereas the treatment of a large TCF is always complicated and can lead to various foreseeable complications (5). Here we reported that the large TCF was successfully treated with bronchoscopic intervention, biodegradable material, and flap grafting, and without cartilage grafting. The patient provided written informed consent to have the case report and any accompanying images published. Institutional approval was obtained from the Institutional Ethical Review Board of the First Hospital of China Medical University to publish the case details.

## Case Presentation

**Figure 1:**
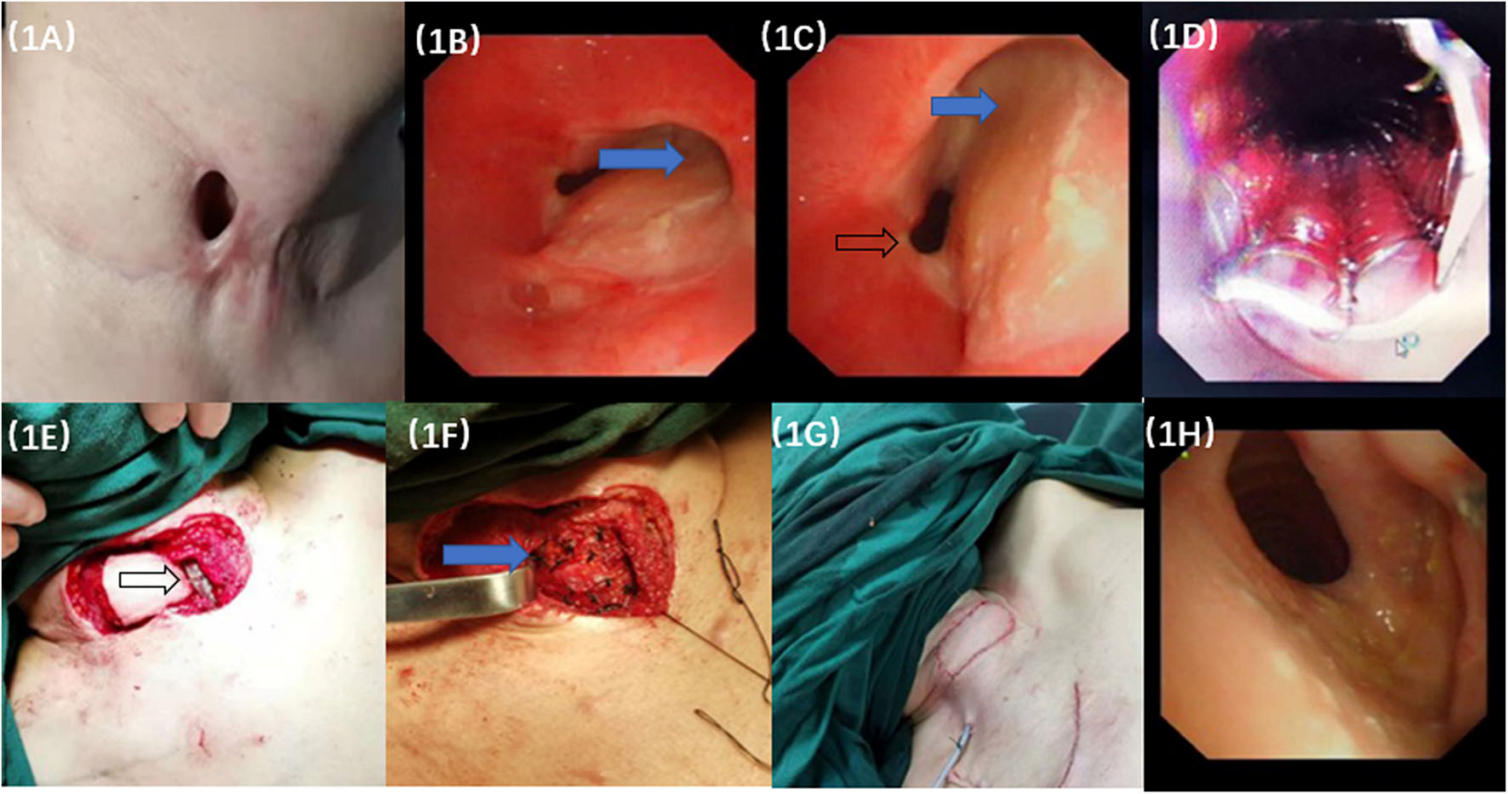
The manifestation of surgery and bronchoscopic images. **(A)** The tracheocutaneous fistula (TCF) measured 2.0 cm × 1.0 cm on the skin to the right side edge of the trachea. **(B,C)** Bronchoscopy revealed a fistula on the right wall of the tracheal, ~1 cm below the glottis, with an area of ~3.0 cm × 1.0 cm. It caused the dynamic compression of the lumen when it was sucked inwards at inspiration (Blue arrow). Black arrow showed trachea lumen. **(D)** The TCF was blocked by the covered airway stent. **(E)** The black arrow showed the airway stent waiting for the cover by the turnover flap. **(F)** The blue arrow showed that the degradable materials were vertically fixed to the grafting flap on the trachea. **(G)** Completion of the flap grafting. **(H)** Repeated bronchoscopy examination 12 months later showed that the anastomosis healed well from the inside view of the trachea lumen without constriction or dynamic stenosis.

A 33-year-old woman was admitted to our hospital with a history of thyroid carcinoma treated with thyroidectomy and partial trachea resection, but a persistent TCF had developed. The stoma measured 2.0 cm × 1.0 cm on the skin to the right side of the trachea (Figure 1A). Bronchoscopy revealed a fistula on the right side of the tracheal wall, ~1 cm below the glottis, with an area of ~3.0 cm × 1.0 cm in size (Figures 1B,C). The edge of the tracheal fistula curled toward the lumen and presented with dynamic compression and stenosis. Three-dimensional computed tomography (3D-CT) of the trachea demonstrated that there was partial absence of the trachea. Given the size and complexity of the TCF, a decision was made to repair the defect using bronchoscopy combined with flap transplantation.

There were four steps in the whole surgical procedure.

1. A temporary covered tracheal stent was transmitted into the tracheal lumen through a rigid bronchoscope, and the stent was rapidly deployed over the stoma. Internal coverage of the entire defect by the tracheal stent was confirmed by flexible bronchoscopy (Figure 1D).
2. The scar and the hyperplastic tissue around the fistula were carefully incised. A turnover flap was made on the right side to cover the fistula. The peripheral flaps were then elevated, and the turnover flap was secured to the healthy skin edge right around the orifice and covered the stoma under optimal tension. The tracheal layer was sutured with interrupted 4-0 Vicryl rigorously (Figure 1E).
3. Two pieces of degradable material (RapidSorb Plate 2.0, Depuy Synthes, Oberdorf, Switzerland) measuring 1.0 cm × 4.0 cm were vertically fixed to the grafting flap on the trachea (Figure 1F).
4. The cervical skin defect measured 5.0 cm × 3.0 cm after tracheal wall reconstruction. A deltopectoral flap measuring 6.0 cm × 4.0 cm in size was dissected and transferred to the cervical area from the pectoral wall under the subcutaneous tunnel. The thoracic defect was closed under appropriate tension with 3-0 Vicryl subcutaneously. Then, 4-0 Monocryl was used to close the external skin tightly. Ventilation was maintained during stent implantation and fistula resection and reconstruction (Figure 1G).

**Figure 2:**
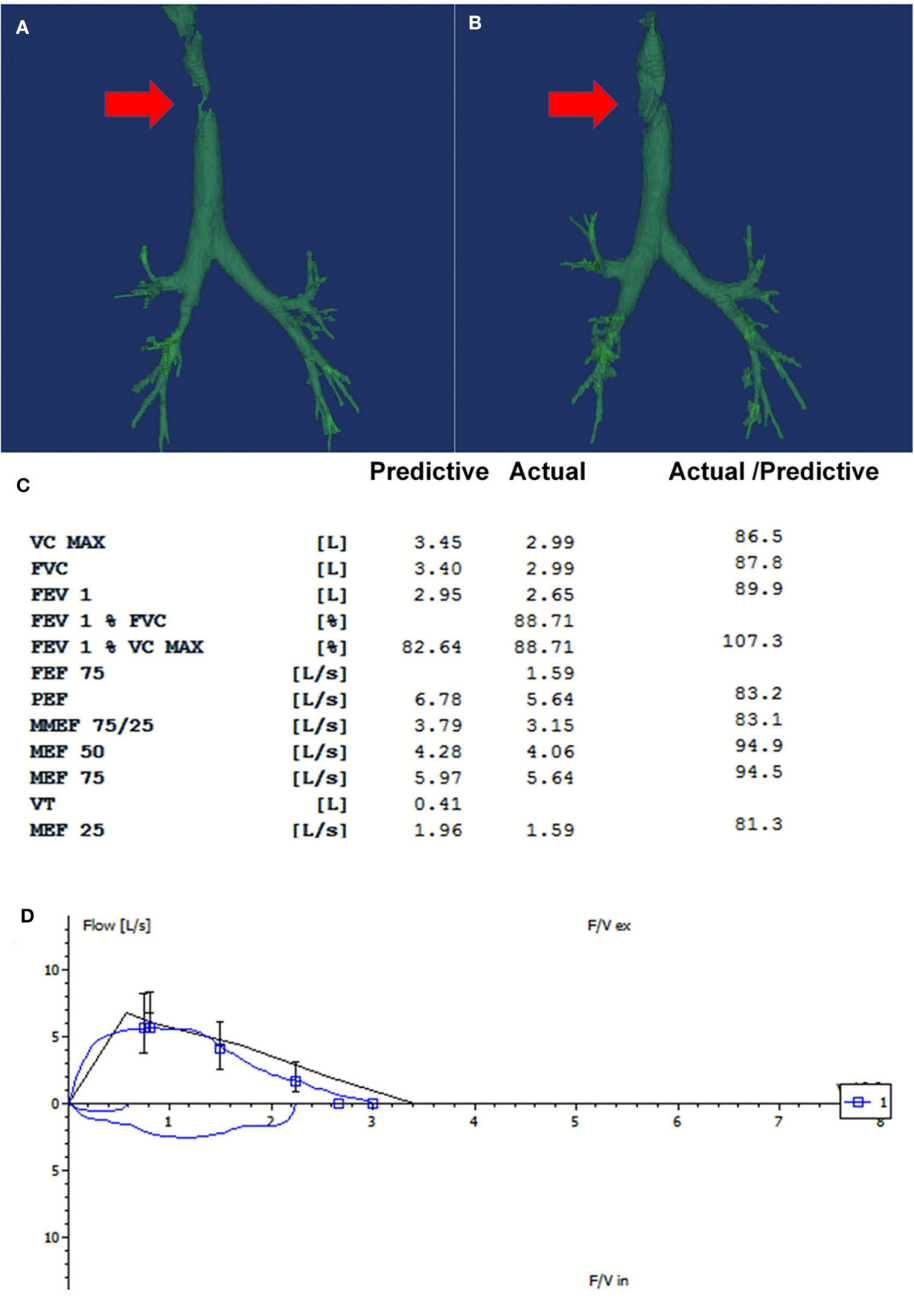
The repeated three-dimensional computed tomography (3D-CT) of the trachea and the spirometry at 16-month follow-up. **(A)** There was partial absence at the right side of the trachea on the 3D-CT on admission (Red arrow pointed to the fistula). **(B)** The repeated CT at 16-month follow-up showed that the fistula was covered by the flap grafting (Red arrow). **(C)** The spirometry at 16-month follow-up showed that the ventilatory function was normal. **(D)** The flow-volume loop showed that no fixed or dynamic airway obstruction existed. Good patency and stableness of large airway was maintained after the treatment.

The tracheal stent did not induce broncho-aspiration or swallowing troubles during the follow-up. The tracheal stent was removed 2 weeks later, and the fistula healed well. Twelve months later, bronchoscopy examination showed that the anastomosis healed well. Mucosal epithelial metaplasia occurred in the lumen with no constriction or dynamic stenosis of the trachea (Figure 1H). The patient reported improvement of the quality of life and vocal function, responded well to the therapy and did not experience recurrence or complications at the 12-month follow-up. The 3D-CT of trachea showed recovery of the TCF, and the spirometry was normal which indicated no fixed or dynamic airflow limitation and the maintain of the patency and stableness of the large airways at the 16-month follow-up (Figure 2).

## Discussion

TCFs are usually iatrogenic. Although not life threatening, the development of a TCF after thyroid tumor excision and tracheostomy is a relatively common long-term bothersome complication (2), but the dimension of the orifice as large as our case is rarely seen. It had largest fistula and higher risk of airway collapsing. A large TCF can induce an increased possibility of respiratory tract bacterial infections, foreign body aspiration, diminished respiratory function, cosmetic defects, and inconvenience when swimming or bathing (1, 5).

For large TCFs, it is necessary not only to complete the skin defect closure but also to reconstruct the missing component of the tracheal wall (2). Some researchers have described management of large TCF. In 2015, Allison K. and Mark C. used a prefabricated radial forearm free flap to cure a large fistula (5). However, this patient had a history of radiation and needed a free flap from the distant flap, whereas ours did not; thus, a turnover flap was enough for a more simple and manageable manipulation. Moreover, the manufacturing operation of a prefabricated composite graft took more than 6 weeks, which immensely increased the possibility of bacterial infections, extra scarring and inconvenience in life. Artificial materials were used successfully for the reconstructive upholder, so there was no need to construct complicated flaps. Takeshi Kitazawa presented a treatment strategy to close a TCF with a local turnover flap combined with pregrafted palatal mucosa (6). However, our case presented more complex dynamic stenosis and marginal cartilage invagination, and simple flap grafting cannot address this clinical scenario. Moreover, their whole procedure consisted of 2 stages, which added the incidence of possible complications such as mucosa infection, surgical emphysema, respiratory distress, bleeding, pneumothorax, and apneic episodes (9). We did not choose sterno-cleido-mastoid muscle flap, although it would be sufficient to repair such soft tissue defect in this case, and it was closer than the delto-pectoralis flap to the defect area. In the very beginning, it was one alternative option in our flap choice. In this case, the patient is a young lady with strong intentions of leaving extra scars in hidden place and restoring better neck function. So we chose delto-pectoralis flap rather than sterno-cledo-mastoid flap for aesthetic and functional perspectives, thus there would be no additional scarring in the exposed neck area. We also did not choose a simple resection and anastomosis of the trachea as option of treatment, because the patient worried about the complications, such as re-constriction of tracheal lumen, anastomotic fistula, the risk of limitation of movement of the head and neck, and refused to accept resection and anastomosis.

The first problem we encountered was that the fringe of cartilage of the trachea wall was invaginated toward the trachea lumen, and the recovery of the radian of the airway must be taken into consideration. General surgical methods by outward flap grafting, such as cartilage grafts (5), palatal mucosal grafts (6), turnover skin flaps (4), cannot perfectly satisfy the restoration of tube wall radians and may exacerbate simultaneous dynamic stenosis and lead to severe complications such as dyspnea, apnea, subcutaneous emphysema, dysphonia, and respiratory failure (10). Therefore, we used the covered metal tracheal stent from the inside of the lumen to reshape the tracheal fistula area by performing temporary covered tracheal stent implantation. The covered stent also provided protection from air inflow leaking out and causing subcutaneous emphysema in case the patient coughed post-operatively. The closure of a TCF is feasible with a temporary covered tracheal stent, and the stent provides an alternative option when coping with these challenging problems.

The second problem was repairing the fistula and avoiding the collapse of flap. Our management strategy was to use a local turnover flap from contiguous skin, followed by stenting the flap with two parallel biodegradable materials. In this way, we can create a rigid framework of the tracheal wall that is needed to avoid collapse of the soft tissue (2). The patient presented with dynamic stenosis that increased the difficulty of TCF reconstruction. External fixation with degradable materials was required to reduce the post-operative dynamic stenosis of the trachea by preventing the flap from protruding into the bronchial lumen during expiration, ensuring the patency of the trachea (11). A few months later, the two patches degraded, and fibrotic tissue came into being. The rigid frameworks for tracheal defects can widely restrain the flap from collapsing. A potential advantage of using degradable materials was avoiding the injury of cartilage grafting (5). But it needs more cases or case control study to be identified.

In conclusion, the management of large TCF is still challenging, the combination of bronchoscopic intervention, biodegradable material, and flap grafting was a potential effective treatment. It offers valuable information for further study in the treatment of large TCFs.

## Ethics Statement

Ethical review and approval was not required for the study on human participants in accordance with the local legislation and institutional requirements. The patients/participants provided their written informed consent to participate in this study. Written informed consent was obtained from the individual(s) for the publication of any potentially identifiable images or data included in this article.

## Author Contributions

GH made substantial contributions to the conception and design of the work. GH, JL, and CL helped to collect the data of the case. GH, HL, and NZ write the manuscript and performed the surgical procedure. GH and HL carried out interpretation of data for the work. All authors revised the paper critically for important intellectual content. All authors carried out final approval of the version to be published. All authors agree to be accountable for all aspects of the work in ensuring that questions related to the accuracy or integrity of any part of the work are appropriately investigated and resolved. All authors contributed toward acquisition of data for the work.

## Conflict of Interest

The authors declare that the research was conducted in the absence of any commercial or financial relationships that could be construed as a potential conflict of interest.

## Abbreviations

TCF: tracheocutaneous fistula.

## References

[1] Drezner DA Cantrell H. Surgical management of tracheocutaneous fistula. Ear Nose Throat J. (1998) 77:534–7. 9693468

[2] Hamahata A Beppu T Yamaki T Sakurai H. Primary reconstructive method for tracheal defect from invasion by differentiated thyroid carcinoma. Auris Nasus Larynx. (2018) 45:371–6. 10.1016/j.anl.2017.05.004 28522300

[3] Khatri R Sarkar S Mehta AR. Management of tracheocutaneous fistula. Indian J Otolaryngol Head Neck Surg. (2001) 53:158–9. 10.1007/BF02991516 23119784 PMC3450857

[4] Lee UJ Goh EK Wang SG Hwang SM. Closure of large tracheocutaneous fistula using turn-over hinge flap and V-Y advancement flap. J Laryngol Otol. (2002) 116:627–9. 10.1258/00222150260171632 12389692

[5] Royer AK Royer MC Ting JY Weisberger EC Moore MG. The use of a prefabricated radial forearm free flap for closure of a large tracheocutaneous fistula: a case report and review of the literature. J Med Case Rep. (2015) 9:251. 10.1186/s13256-015-0728-z 26520064 PMC4628779

[6] Kitazawa T Shiba M. Closure of a tracheocutaneous fistula with a local turnover flap combined with pregrafted palatal mucosa: a case report. Eplasty. (2016) 16:e30. 27980701 PMC5120373

[7] Goldsmith AJ Abramson AL Myssiorek D. Closure of tracheocutaneous fistula using a modified cutaneous Z-plasty. Am J Otolaryngol. (1993) 14:240–5. 10.1016/0196-0709(93)90066-g 8214315

[8] Fisher SR. Closure of tracheocutaneous fistula with perichondrial flap following cricothyroidotomy. Laryngoscope. (1991) 101(6 Pt 1):684–5. 10.1288/00005537-199106000-00020 2041452

[9] Geyer M Kubba H Hartley B. Experiences of tracheocutaneous fistula closure in children: how we do it. Clin Otolaryngol. (2008) 33:367–9. 10.1111/j.1749-4486.2008.01729.x 18983350

[10] Lewis S Arjomandi H Rosenfeld R. Systematic review of surgery for persistent pediatric tracheocutaneous fistula. Laryngoscope. (2017) 127:241–6. 10.1002/lary.26080 27175967

[11] Baste JM Haddad L Philouze G. A combined technique using a muscular flap and endobronchial stent to repair complex broncho-oesophageal fistulae supported by ECMO. Acta Chir Belg. (2018) 118:52–5. 10.1080/00015458.2017.1284466 28685656

